# Synergistic waste-derived fillers induce polar phase transformation for high-performance fluorinated polymer

**DOI:** 10.1039/d5ra09097k

**Published:** 2026-02-10

**Authors:** Islam Gomaa, Fatma Gamal, Haitham Kalil, Hanan Elhaes, Ahmed I. Ali, Dongwhi Choi, Galal H. Ramzy, Medhat A. Ibrahim

**Affiliations:** a Nanotechnology Research Centre (NTRC), The British University in Egypt (BUE) Suez Desert Road El-Sherouk City Cairo 11837 Egypt; b Department of Basic Sciences, October High Institute of Engineering and Technology - OHI 6th of October City Giza Egypt; c Physics Department, Faculty of Women for Arts, Science and Education, Ain Shams University 11757 Cairo Egypt hanan.elhaes@women.asu.edu.eg; d Department of Chemistry, Suez Canal University Ismailia Egypt; e Department of Basic Science, Faculty of Technology and Education, Capital University (Formerly Helwan University) Saray El-Quba 11281 Cairo Egypt ahmed_ali_2010@techedu.helwan.edu.eg; f Department of Mechanical Engineering (Integrated Engineering Program), Kyung Hee University 1732 Deogyeong-Daero Yongin Gyeonggi 17104 Republic of Korea dongwhi.choi@khu.ac.kr; g Physics Department, Faculty of Science, Cairo University Giza 12613 Egypt; h Spectroscopy Department, National Research Centre 33 El-Bohouth St. 12622 Dokki Giza Egypt; i Molecular Modeling and Spectroscopy Laboratory, Centre of Excellence for Advanced Science, National Research Centre 33 El-Bohouth St. 12622 Dokki Giza Egypt

## Abstract

This manuscript reports a scalable, solution-cast strategy to upcycle waste-derived functional fillers into electroactive poly(vinylidene fluoride) (PVDF) hybrid membranes for promising dielectric/piezoelectric applications. Carbon-doped recycled ZnO (Rc-ZnO) microspheres are synthesized from spent zinc–carbon batteries and combined with graphene oxide (GO) to fabricate PVDF/Rc-ZnO, PVDF/GO, and ternary PVDF/Rc-ZnO/GO films at a fixed total loading of 10 wt% (5 wt% Rc-ZnO/5 wt% GO in the ternary system) using DMF/acetone casting and controlled thermal drying. Structural and spectroscopic analyses (XRD/FT-IR/SEM) confirm a filler-driven α → β transformation, where GO defect sites act as nucleation centers and the dual-filler system increases the β-phase fractions alongside a morphology evolution toward a more interconnected porous architecture. Dielectric spectroscopy shows that co-doping delivers the most favorable electrical response, with AC conductivity increasing with frequency and temperature due to synergistic charge-carrier generation by ZnO and conductive pathways provided by GO. Density-functional-theory calculations (B3LYP/DGDZVP2) further rationalize these trends by revealing a strong reduction in the electronic gap upon interfacial hybridization (PVDF: 9.251 eV; GO/PVDF/ZnO: 1.595 eV; d-GO/PVDF/ZnO: 0.833 eV), supporting enhanced interfacial polarization and charge transport.

## Abstract 2

This manuscript reports a scalable, solution-cast strategy to upcycle waste-derived functional fillers into electroactive poly(vinylidene fluoride) (PVDF) hybrid membranes for promising dielectric/piezoelectric applications.

## Graphical abstract

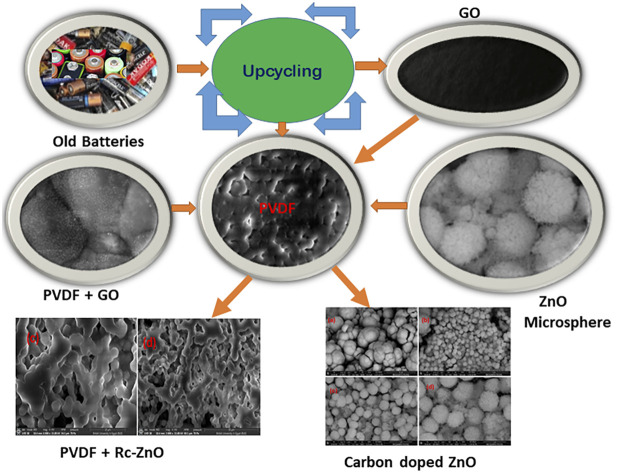

## 1 Introduction

Poly(vinylidene fluoride) (PVDF) is among the most technologically mature electroactive polymers because it combines chemical/thermal stability with processability into thin films and membranes for flexible electronics, sensing, and energy harvesting.^1,2^ Dielectric properties of PVDF especially the frequency/temperature dependence of permittivity (*ε*′), dielectric loss (tan *δ*), and relaxation features, directly quantify the strength and dynamics of dipolar and interfacial polarization mechanisms that control charge storage, dissipation, and extractable electrical response in electroactive polymer films and membranes.^3,4^

Its electromechanical performance, however, is strongly phase-dependent: the polar β-phase (all-trans conformation) provides the highest dipole alignment and therefore the most desirable piezoelectric/ferroelectric response, whereas the α-phase is essentially non-polar and limits device-level output.^5^ In practical solution-cast PVDF, achieving a high and stable β-phase fraction remains nontrivial because crystallization kinetics, solvent–polymer interactions, and chain mobility typically favor mixed polymorph populations unless additional driving forces are introduced.^6^ Waste-derived metal oxides and carbon nanostructures can serve not only as reinforcing additives, but also as electro-crystallization templates that promote the electroactive β-phase and improve dielectric functionality.^7^ ZnO offers a rare electronic “sweet spot” for functional composites,^8^ its wide direct band gap (∼3.37 eV) and unusually large exciton binding energy (∼60 meV) enable strong near-band-edge optical/electronic activity that remains stable at room temperature,^9^ highly advantageous for charge-generation/charge-separation roles in hybrid materials and electroactive membranes.^10^

Nowadays, sustainable and scalable ZnO supply, especially routes that convert zinc-rich wastes into device-grade ZnO with reproducible electronic properties remains relatively fragmented compared with conventional precursor-based synthesis: although several studies demonstrate recovery/synthesis of ZnO nanoparticles from spent Zn–C or alkaline batteries *via* thermal and hydrometallurgical/green upcycling pathways,^11^ they are still scattered across recycling and nanomaterials domains and rarely unified into standardized, quality-controlled workflows.^12^ This gap strongly motivates establishing a coordinated extraction/valorization network (collection → disassembly → purification → conversion → QC) to ensure that waste-derived ZnO can be produced at scale with consistent electronic performance suitable for advanced PVDF composite membranes.^13^ Hydrometallurgical techniques offer selective Zn-extraction with high purity accompanied by low yield,^14^ and bioleaching, an emerging eco-friendly method, uses microorganisms to extract zinc under mild conditions. Once extracted, zinc is converted into ZnO NPs using methods like precipitation sol–gel synthesis, or green synthesis, with each technique offering specific advantages in terms of cost, scalability, and nanoparticle properties.^15–17^ Despite the promising potential, challenges such as optimizing extraction processes, improving synthesis scalability, and assessing environmental impacts remain. Nonetheless, this approach supports waste reduction and aligns with circular economy principles, highlighting its importance for sustainable resource management.^18^ Adaval *et al.* (2022) developed PVDF/GO nanocomposites optimized for piezoelectric applications, where the incorporation of functionalized GO nanosheets markedly boosted the output voltage to 8.5 V, significantly exceeding that of pure PVDF.^19^ Amina *et al.* (2023) conducted a pivotal study on the synergistic doping of ZnO nanosheets and reduced graphene oxide (rGO) in PVDF using solution casting to enhance its β-phase, thereby boosting its piezoelectric properties.^20^ Singh and Khare (2024) engineered a piezo-tribo hybrid generator by integrating ZnO with PVDF and PTFE, resulting in a significant enhancement of triboelectric properties. The composite achieved a maximum output power of approximately 24.5 µW cm^−2^, which is 2.5 times higher than that of a pure PVDF-based generator.^21^ Alshahrani *et al.* (2024) demonstrated that the incorporation of ZnO into PVDF composites significantly enhances the β-phase, thereby improving piezoelectric performance.^22^

In this work, we combine sustainable materials valorization with a mechanistically grounded structure–property analysis by engineering a GO/PVDF/ZnO composite (with emphasis on waste-to-ZnO scalability) and quantitatively linking its electroactive response to interfacial electronic descriptors. Beyond reporting phase and morphology, we integrate XRD, FT-IR, and FE-SEM with dielectric spectroscopy to establish how ZnO/GO interfaces promote electroactive PVDF ordering and govern polarization/loss behavior under alternating fields. To close the interpretation gap between microstructure and function, DFT calculations at the B3LYP/DGDZVP2 level are used to extract total dipole moment, computed HOMO/LUMO gap, and density of states, enabling an evidence-bounded explanation of the observed dielectric/electroactive trends and providing design rules for scalable PVDF-based multifunctional device-level proof-of-concept.^23^

## 2 Materials and methods

### 2.1. Chemicals and reagents

All chemicals were used as received without further purification. Discharged commercial zinc-carbon batteries (Eveready, Egypt) were collected from local waste streams/recycling bins and used as the Zn source for preparing recycled ZnO. Citric acid (99%, Fisher Chemical), poly(vinylidene fluoride) (PVDF; Mw ≈ 180 000 g mol^−1^ by GPC), *N*,*N*-dimethylformamide (DMF; Mw 73.09 g mol^−1^, Fisher Chemical), acetone (99%, Fisher Chemical), ammonia solution (35%, Fisher Chemical), sodium hydroxide (≥97%, Fisher Chemical), and graphite powder (Fluka, Germany) were used as received. Sulfuric acid (H_2_SO_4_, 98%), hydrochloric acid (HCl, 33%), and hydrogen peroxide (H_2_O_2_, 30%) were purchased from El-Nasr Pharmaceutical Company (Cairo, Egypt), and potassium permanganate (KMnO_4_, 98%, Alfa Aesar, Germany) was employed for GO synthesis. Deionized Milli-Q water was used throughout all experiments.

### 2.2. Synthesis of graphene oxide sheets

Regarding to prior well established studies.^24,25^ For the preparation of graphite oxide, 1 g of graphite powder was added to 23 mL of concentrated H_2_SO_4_ in a reaction vessel placed in an ice bath to maintain the temperature below 20 °C. Gradually, 3 g of KMnO_4_ was added to the mixture while stirring continuously, ensuring slow addition to prevent overheating. The mixture was then stirred at 35 °C for 2 hours to ensure complete oxidation. After this period, 46 mL of distilled water was added slowly, and the temperature was raised to 98 °C and maintained for 15 minutes. The reaction was terminated by adding 140 mL of distilled water followed by 10 mL of 30% H_2_O_2_, turning the color of the mixture from dark to dark brown, indicating the formation of graphene oxide (GO). The resulting mixture was filtered and washed with distilled water until the pH of the filtrate became neutral.^26^

### 2.3. Synthesis of ZnO microsphere

**Scheme 1:**
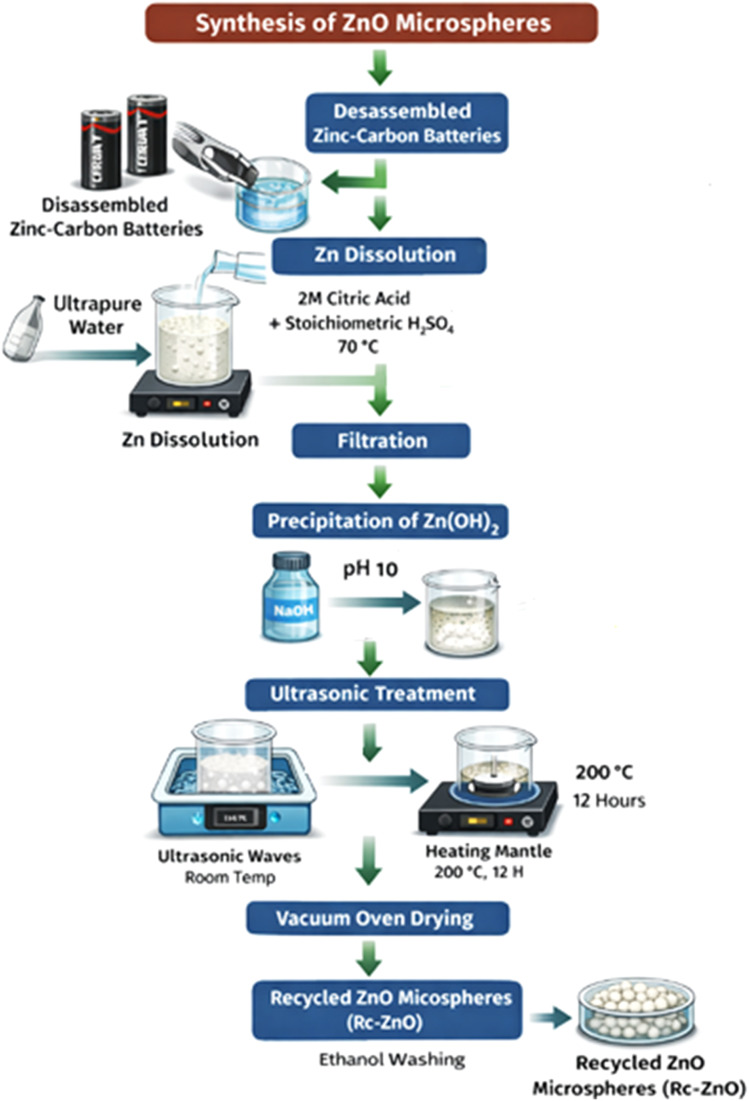
Schematic synthesis of carbon-doped recycled ZnO (Rc-ZnO) microspheres from spent zinc–carbon batteries.

Leveraging insights from prior research (Scheme 1)^27–29^ on optimizing synthesis conditions, this work presents a facile approach for the fabrication of ZnO nanoparticles (NPs). Disassembled zinc–carbon batteries served as the zinc source. The zinc casing was mechanically removed and meticulously washed with ultrapure water to eliminate any potential contaminants. Subsequently, the zinc was dissolved in a solution composed of 2 M citric acid and stoichiometric ratio of H_2_SO_4_ under vigorous stirring at 70 °C until complete dissolution was achieved. Filtration was then employed to remove any residual undissolved particulates. Following the dissolution step, precursor formation Zn(OH)_2_ was undertaken. The zinc-rich solution was transferred to a reaction vessel and a 2 M NaOH solution was gradually added under constant stirring until the pH reached approximately 10. This resulted in the precipitation of a white Zn(OH)_2_ product. The mixture was further stirred for 30 minutes to ensure complete precipitation. The Zn(OH)_2_ precipitate was isolated *via* filtration and rigorously washed with ultrapure water to eliminate any remaining NaOH residues. To achieve homogenous dispersion, the Zn(OH)_2_ precipitate was resuspended in ultrapure water and subjected to ultrasonic treatment in a bath for 30 minutes. The suspension was then transferred to a heating mantle and maintained at 200 °C for 12 hours under continuous stirring in vacuum oven.^30^ This thermal treatment facilitated the dehydration of Zn(OH)_2_, leading to the formation of ZnO NPs. Finally, the NPs were thoroughly washed with ethanol to eliminate any adsorbed organic impurities and dried at 60 °C overnight at vacuum oven. Obtained off-white powder as recycled ZnO nominated as Rc-ZnO.

### 2.4. Fabrication of PVDF, PVDF/ZnO, PVDF/GO and PVDF/ZnO/GO hybrid membranes

**Scheme 2:**
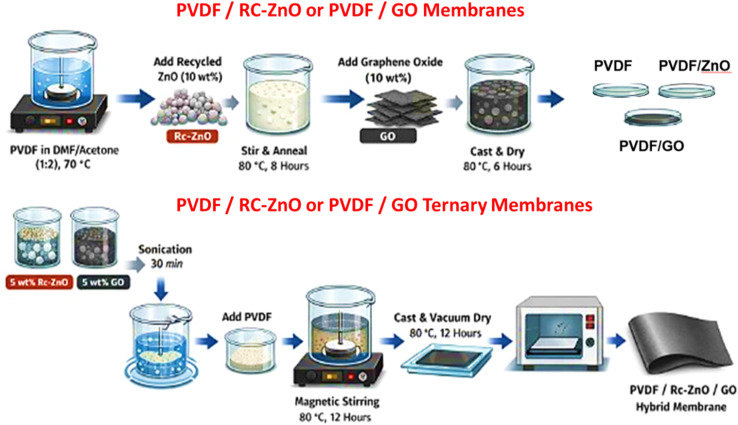
Schematic illustration of the step-by-step fabrication procedure for PVDF, PVDF/Rc-ZnO, PVDF/GO, and PVDF/Rc-ZnO/GO hybrid membranes.

PVDF (3.0 g) was dissolved in a mixed solvent system of DMF/acetone (1 : 2, v/v) to obtain a 5 wt/v% casting solution. Dissolution was carried out under stirring at 70 °C to ensure complete polymer solvation and to minimize undissolved crystallites that can act as unintended nucleation centers during subsequent recrystallization and film formation.^30^ After obtaining a clear PVDF solution, recycled ZnO (Rc-ZnO) or GO was added separately at a total loading of 10 wt% (relative to PVDF) and dispersed by continuous stirring, followed by thermal conditioning at 80 °C for 8 h to enhance dispersion uniformity and promote controlled chain relaxation prior to casting. The resulting dispersions were cast onto clean Petri dishes and dried at 80 °C for 6 h to remove solvents and yield flexible self-standing membranes, denoted PVDF/Rc-ZnO and PVDF/GO. For the ternary PVDF/Rc-ZnO/GO membrane, the total filler loading was maintained at 10 wt% and distributed as 5 wt% Rc-ZnO microspheres +5 wt% GO. Rc-ZnO and GO were first co-dispersed in the DMF/acetone mixture using bath sonication (30 min, continuous mode) to promote deagglomeration and homogeneous filler distribution. PVDF was then added to the suspension and the mixture was magnetically stirred at 80 °C for 12 h to strengthen filler-polymer interfacial interactions and ensure stable viscosity prior to casting. The nanocomposite solution was cast onto a clean glass substrate and dried in a vacuum oven at 80 °C for 12 h (Scheme 2). The longer dwell time for the ternary formulation was employed because the dual-filler suspension exhibits higher viscosity and stronger filler–filler/filler–polymer interactions, which slow solvent diffusion; extended annealing was therefore required to reach constant mass, stable thickness, and reproducible dielectric measurements.

### 2.5. Characterization techniques

Fourier transform infrared (FT-IR) spectroscopy was employed to investigate the chemical functionalities of the samples. The measurements were conducted using an Attenuated Total Reflection (ATR) accessory on a Vertex 70 spectrometer (Bruker, Germany). The spectra were acquired within a wavenumber range of 4000–400 cm^−1^ with a resolution of 4 cm^−1^. X-ray diffraction (XRD) analysis was performed to elucidate the phase composition and crystallographic structure of the as-prepared samples. A Malvern Panalytical Empyrean 3 diffractometer was utilized for this purpose. This technique provided high-resolution images of the surface features. Furthermore, Scanning Electron Microscopy (SEM) (SEM-QUANTA FEG-250) was employed to characterize the morphology and elemental composition of the samples at high magnification.

### 2.6. Dielectric properties

Dielectric permittivity and loss tangent of the samples as a function of frequency and temperature were carried out using an impedance analyzer (HP4192). A magnetron sputtering system (KVS C4055) was used to deposit gold (40 nm) on the nanocomposites membranes as bottom and top contact. The following relation is used to calculate the real part of dielectric constant (*ε*′):1
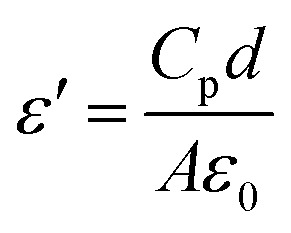
where, *C*_p_ is the capacitance, *d* is the thickness of the membrane, *A* is the membranes' cross section area, and *ε*_o_ is the permittivity of free space (8 × 10^−12^). The energy loss in a dielectric material through conduction in the form of heat is called dielectric loss (*ε*′′). The following relation is used to determine the dielectric loss value for the prepared samples:

2 ε″ = *ε*′ tan *δ*

where, tan(*δ*) is the dielectric loss angle (dissipation factor) which is determined from an impedance analyzer (HP4192). Further, AC conductivity (*σ*_AC_) of the samples can be calculated from the values of real part of dielectric constant and loss tangent using the following equation:

3 *σ*_AC_ = 2π*fε*_o_*ε*′ tan *δ*

where *f* is the frequency of the applied electric field during the measurements.^31^

### 2.7. Calculation details

**Fig. 1:**
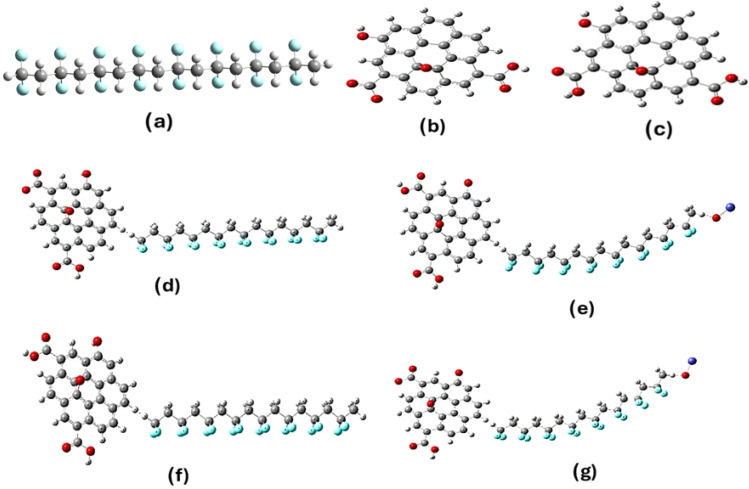
Model molecules for the main structures in the present study whereas (a) PVDF, (b) deprotonated GO (d-GO), (c) protonated GO (GO), (d) GO/PVDF, (e) GO/PVDF/ZnO, (f) GO/PVDF and (g) d-GO/PVDF/ZnO which calculated at B3LYP/DGDZVP2.

Model molecules for PVDF; protonated graphene oxide GO, and deprotonated graphene oxide GO are indicated in Fig. 1. PVDF is supposed to interact with GO; GO and ZnO through van der Waals interaction forming GO/PVDF/ZnO and GO/PVDF/ZnO as indicated also in Fig. 1. The studied molecules were subjected to optimization at density functional theory method DFT; B3LYP methods,^23^ together with DGDZVP2 basis set. Calculations were conducted with Gaussian 09^32^ soft code which was implemented at National Research Centre. Total dipole moment TDM, HOMO/LUMO energy gap were calculated then the density of states DOS were mapped at the same level of theory.

## 3 Results and discussion

### 3.1. X-ray diffraction analysis

**Fig. 2:**
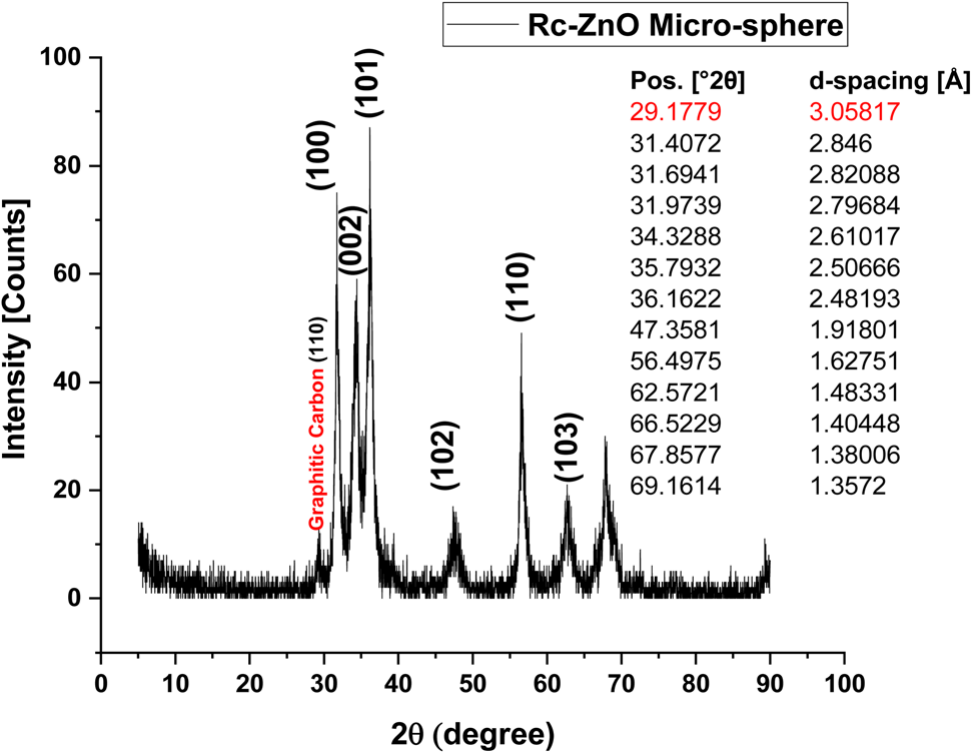
X-ray diffraction (XRD) patterns of ZnO micro sphere.

Prolonged drying at 200 °C resulted in the formation of a powder, as confirmed by powder X-ray diffraction (XRD) analysis in Fig. 2. The diffraction patterns revealed distinct peaks corresponding to ZnO and residual carbon. Peaks at 2*θ*: 31.97°, 34.33°, 35.79°, 36.16°, 47.36°, 56.49°, 62.57°, 66.52°, 67.86°, and 69.16° were consistent with the hexagonal crystal structure of ZnO, as referenced by card number 01-075-0576. The corresponding *d*-spacing values for these peaks are 3.058 Å, 2.846 Å, 2.821 Å, 2.797 Å, 2.617 Å, 2.506 Å, 2.482 Å, 1.918 Å, 1.627 Å, 1.483 Å, 1.404 Å, 1.380 Å, and 1.357 Å, respectively. Notably, peaks at 2*θ* positions of 29.18°, 31.41°, and 31.69° were indexed to carbon monoxide, aligning with reference card 01-082-5764.^33–35^

**Fig. 3:**
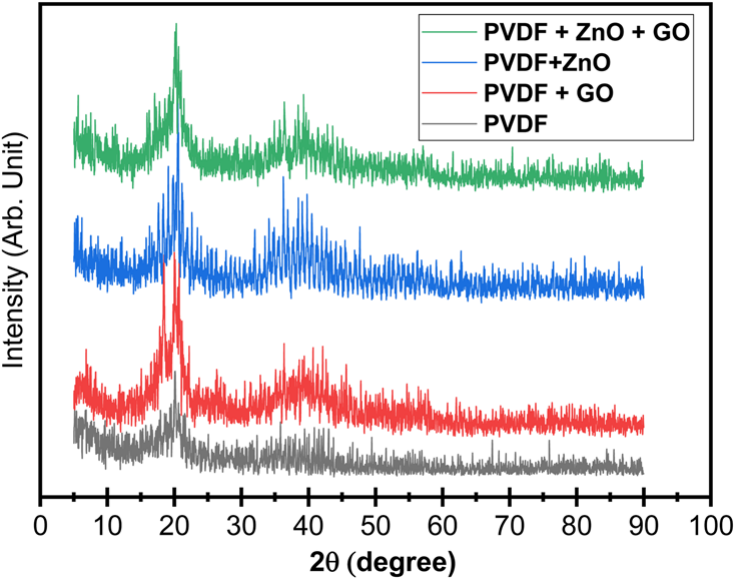
X-ray diffraction (XRD) patterns of PVDF, PVDF/GO, PVDF/ZnO, and PVDF/ZnO/GO membranes.

XRD patterns of the commercial PVDF (Fig. 3) show the characteristic α-phase reflections at 2*θ* ≈ 18.6° (020) and 19.9° (110), with a higher-angle feature around 26.4° (021), confirming that the pristine sheets are predominantly α-crystalline.^42^ In addition, the diffractograms display broad semi-amorphous halos (≈17.9–26.0° and 30–45°), evidencing the coexistence of crystalline and amorphous domains typical of PVDF.^19^ Upon membrane formation and filler incorporation, the diffraction profile evolves: the α-phase reflections broaden and lose intensity, while a peak centered at 2*θ* ≈ 20.2–20.4° becomes more pronounced, consistent with the emergence of the electroactive β-phase.^36,37^ Minor overlap with neighboring α/γ contributions can persist because PVDF polymorph reflections are close in angle, but the strengthened ∼20.4° feature together with the suppressed α markers indicates a clear α → β enrichment in the hybrid membranes.^38,39^ This phase evolution is consistent with a filler-driven conformational bias, where polar GO sheets and ZnO-containing domains promote dipole–dipole/electrostatic interactions with PVDF's C–F dipoles and favor the all-trans chain conformation associated with β-phase crystallization, providing the structural basis for the enhanced electrical relevant properties.^40–44^

### 3.2. Fourier transform infrared spectroscopy

**Fig. 4:**
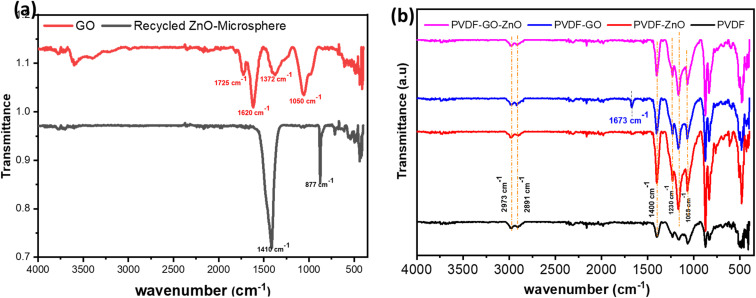
The FT-IR transmittance spectra for (a) recycled ZnO nanoparticles (Rc-ZnO) and graphene oxide (GO), and (b) PVDF, PVDF-Rc-ZnO, PVDF-ZnO-GO, and PVDF-ZnO-GO hybrid membranes.

The FT-IR spectra (Fig. 4a and b) validate both the successful synthesis/functionalization of the fillers and the structure–property pathway that underpins the electrical response of the hybrid membranes. ZnO (embedded in the doped carbon microspheres) shows the unexpected Zn–O surface phonon vibrations at 1410 and 877 cm^−1^ are attributed to trace carbon/graphitic remnants (C–H deformation/C–C stretching) from the carbon precursor,^45,46^ but do not compromise the dominant Zn–O framework in the 400–700 cm^−1^ region (∼445–510 cm^−1^), consistent with a preserved wurtzite lattice and in agreement with the XRD-confirmed crystallinity.^47^ GO displays the canonical oxygenated-carbon fingerprints at ∼1050 cm^−1^ (C–O, epoxy/alkoxy), ∼1372 cm^−1^(C–OH deformation), ∼1620 cm^−1^ (sp^2^ C=C skeletal vibration), and ∼1725 cm^−1^ (C=O stretching), with a broad O–H band (∼3570 cm^−1^),^48^ confirming extensive oxidation/hydroxylation that enhances interfacial compatibility with PVDF. Critically, the membrane spectra (Fig. 4b) evidence a filler-driven α → β phase transition: neat PVDF shows α-phase bands at 484 cm^−1^ (CF_2_ bending), 611 cm^−1^ (CF_2_/skeletal bending), and 870 cm^−1^ (CH_2_ rocking), whereas GO/ZnO incorporation suppresses these α markers and strengthens electroactive features at 840 cm^−1−1^ (β-phase CH_2_ rocking) and 1275 cm^−1^ (β-phase CF_2_ stretching) alongside reinforced bands at 1068, 1167, and 1230 cm^−1^, consistent with increased chain ordering toward all-trans conformations. The selective appearance of a 1673 cm^−1^ band in PVDF-GO further supports strong polymer-GO interfacial coupling (perturbed sp^2^/C=O environment), while the attenuation of ZnO's FT-IR signatures is expected for deeply embedded inorganic domains beyond the effective ATR penetration depth.

### 3.3. FE-SEM

**Fig. 5:**
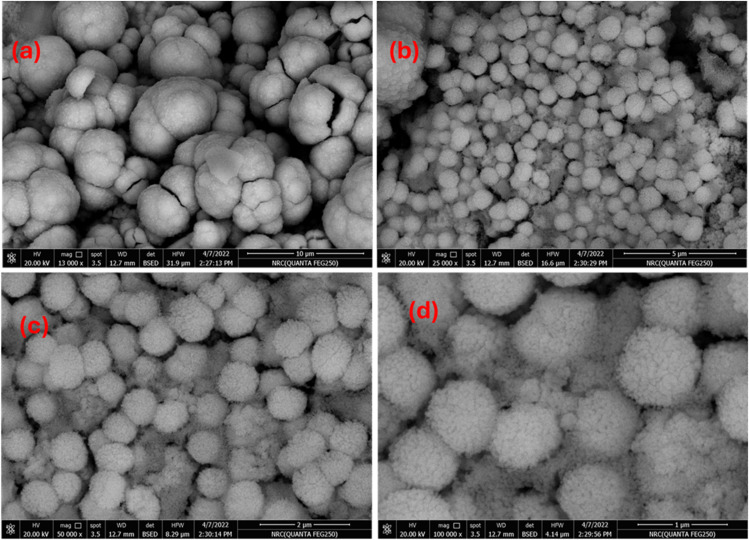
FE-SEM images of Recycled ZnO nanoparticles (Rc-ZnO) assembled in hierarchical micro spherical structure at different magnifications.

Fig. 5 presents a comprehensive FE-SEM analysis of the synthesized ZnO microspheres. At 130 00× magnification (Fig. 5a, scale bar = 10 µm), the images reveal a high density of microspherical ZnO particles with diameters ranging from approximately 5 to 15 µm. These microspheres display a pronounced rough surface texture and evident agglomeration, with larger spheres comprising smaller nanoscale subunits, thereby forming a well-defined hierarchical architecture. At 250 00× magnification (Fig. 5b, scale bar = 5 µm), the surface roughness is even more distinct as the microspheres are enshrouded by a densely packed layer of smaller ZnO nanoparticles, creating a porous morphology that is highly favorable for catalytic and sensing applications due to the increased active surface area. Further magnification at 500 00× (Fig. 5c, scale bar = 2 µm) allows for clear visualization of individual nanoparticles, which are spherical, uniformly distributed, and exhibit diameters between 50 and 200 nm, underscoring their potential for high catalytic activity through a greater density of active sites. At 100 000× magnification (Fig. 5d, scale bar = 1 µm), the intricate arrangement of the nanoparticles becomes evident, with tightly packed particles and well-defined boundaries that suggest enhanced mechanical stability and structural integrity-critical attributes for electronic applications. To conclude, the FE-SEM observations confirm that the ZnO microspheres possess a robust hierarchical structure characterized by high surface area, porosity, and mechanical resilience.

**Fig. 6:**
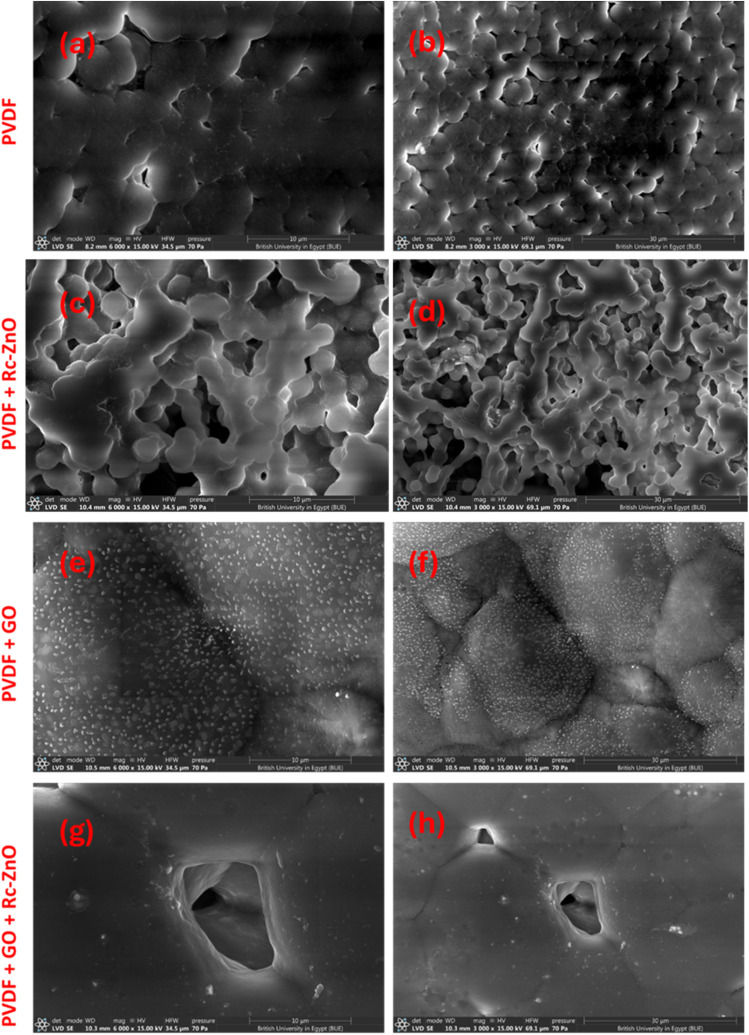
FE-SEM images illustrate the surface morphology of (a and b) PVDF, (c and d) PVDF-Rc-ZnO, (e and f) PVDF-ZnO, and (g and h) PVDF-GO-Rc-ZnO hybrid membranes.

Fig. 6 presents a systematic FE-SEM investigation of PVDF and its hybrid membranes, revealing how nanoscale modifications critically govern structural and functional evolution. Fig. 6(a) and (b) shows the pure PVDF exhibits a dense, compact morphology with localized surface irregularities and micro cracks, characteristic of its semi-crystalline nature. While this architecture ensures mechanical robustness, its low porosity and smooth surface inherently restrict permeability and interfacial activity, a fundamental limitation in filtration or catalytic applications.^41^

The integration of recycled zincite (Rc-ZnO) into PVDF as in Fig. 6(c) and (d) induces a striking morphological transition, producing a heterogeneous, porous matrix with uniformly dispersed ZnO nanoparticles. This nanoscale roughening, observed at both 3000× (30 µm scale) and 6000× (10 µm scale) magnifications, amplifies surface area and introduces hierarchical porosity, which synergistically enhances hydrodynamic interactions and catalytic site accessibility. Such structural tailoring directly addresses PVDF's intrinsic permeability-performance trade-off.^49^

Graphene oxide (GO) incorporation as in Fig. 6(e) and (f), further refines the membrane's architecture, with exfoliated GO sheets intercalated within the PVDF matrix. The textured, layered morphology not only augments hydrophilicity but also reinforces mechanical integrity through GO's stress-transfer capabilities. Crucially, the hybrid PVDF-GO-Rc-ZnO membrane presented in Fig. 6(g) and (h) demonstrates a synergistic interplay: ZnO nanoparticles nucleate at GO defect sites, generating a 3D-interconnected porous network with uniform pore distribution (10–30 µm scale). This hierarchical porosity maximizes surface area while maintaining structural cohesion, enabling simultaneous enhancements in filtration flux, antifouling behavior, and catalytic efficiency.

The observed morphological progression-from PVDF's dense baseline to the hybrid's multifunctional architecture-directly correlates with performance metrics. The Rc-ZnO/GO co-incorporation achieves a critical balance: GO provides mechanical reinforcement and interfacial activation, while ZnO introduces catalytic functionality and pore templating. This combinatorial strategy overcomes PVDF's inherent limitations, positioning the hybrid membrane as a versatile platform for advanced applications requiring high throughput, selective separation, and reactive surface engineering. Future work will quantify structure property relationships through pore-size distribution analysis and nano-indentation, further elucidating design principles for next-generation nanocomposite membranes.

### 3.4. Molecular modeling

**Fig. 7:**
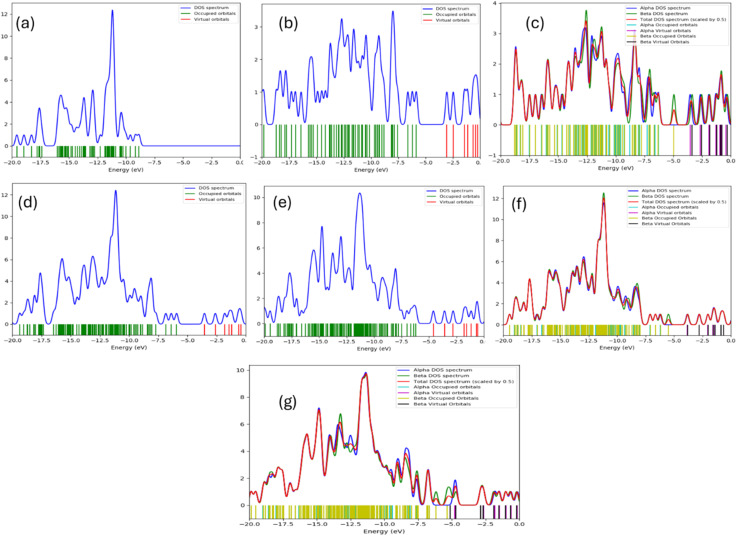
Density of States (DOS) for the primary structures investigated: (a) PVDF, (b) graphene oxide (GO), (c) deprotonated GO (d-GO), (d) GO/PVDF composite, (e) GO/PVDF/ZnO composite, (f) d-GO/PVDF composite, and (g) d-GO/PVDF/ZnO composite, computed at the B3LYP/DGDZVP2 level.

Molecular modeling was conducted to describe the mechanism of interaction between PVDF, GO, and ZnO. The formation of the composite studied could be aided by the molecular modeling in terms the model molecule described in Fig. (7). The models in Fig. (7) describe the main structures in the present study whereas (a) PVDF, (b) deprotonated GO, (c) protonated GO, (d) GO/PVDF, (e) GO/PVDF/ZnO, (f) GO/PVDF, and (g) d-GO/PVDF/ZnO. To describe the physical features of the studied composite both the total dipole moment and HOMO/LUMO energy gap were calculated and listed in Table 1 for the studied structures. For a given chemical composite increasing the values for TDM with decreasing HOMO/LUMO energy could be correlated with the composite reactivity as stated earlier.^50^ As indicated in Table 1, the TDM of PVDF was 16.796 debye while the Δ*E* is also high 9.251 eV. GO shows TDM and Δ*E* as 3.868 debye and 2.799 eV respectively. GO as compared with GO shows a bit higher TDM (10.849 debye) and lower Δ*E* (1.334 eV). This indicated that GO is more reactive than GO. Both GO/PVDF and GO/PVDF/ZnO composites are reactive in terms of the calculated TDM and Δ*E* values. The values for both GO/PVDF and GO/PVDF/ZnO are more reactive as their Δ*E* became 0.025 eV and 0.833 eV. Correlating the results, d-GO enhances the reactivity of PVDF more than GO. To confirm this finding it is important to follow up the molecular orbitals for PVDF as far as interacts with d-GO and GO as well. This could be studied with the help of density of states (DOS) as in the following section.

**Table 1:** Total dipole moment TDM as Debye and HOMO/LUMO band gap energy Δ*E* as eV for the studied structures PVDF, GO, d-GO, GO/PVDF, GO/PVDF/ZnO, d-GO/PVDF and d-GO/PVDF/ZnO which calculated at B3LYP/DGDZVP2

Structure	TDM	Δ*E*
PVDF	16.796	9.251
GO	3.868	2.799
d-GO	10.849	1.334
GO/PVDF	13.595	1.519
GO/PVDF/ZnO	15.172	1.595
d-GO/PVDF	12.241	0.025
d-GO/PVDF/ZnO	16.199	0.833

### 3.5. Density of states (DOS)

The density of states (DOS) is a fundamental descriptor of the electronic structure, quantifying the number of electronic states available per unit energy interval.^51^ Pure PVDF (Fig. 7a) exhibits fully occupied valence states and a very large HOMO–LUMO gap (Δ*E* = 9.251 eV), consistent with its intrinsically insulating response and the dominance of bound-dipole polarization only. In contrast, GO (Fig. 7b; Δ*E* = 2.799 eV) introduces accessible unoccupied states and a far narrower gap, enabling interfacial charge accumulation/hopping that manifest experimentally as the strong low-frequency enhancement of *ε*′ (where interfacial/space-charge polarization can follow the field) and the expected decay of *ε*′ with frequency as these slow processes fall out of phase. Deprotonation is the key electronic “switch”: d-GO (Fig. 7c; Δ*E* = 1.334 eV) shows a marked increase of occupied/virtual-state density across α/β spin channels, *i.e.*, stronger charge delocalization and more transfer-ready states, which explains why d-GO–containing hybrids are predicted to have the most facile carrier dynamics (d-GO/PVDF Δ*E* = 0.025 eV; d-GO/PVDF/ZnO Δ*E* = 0.833 eV). When GO (or d-GO) is embedded in PVDF (Fig. 7d and f) and further coupled with ZnO (Fig. 7e and g), new interfacial states appear and the effective gap collapses relative to PVDF, while the total dipole moment remains high (*e.g.*, GO/PVDF TDM 13.595 D, GO/PVDF/ZnO 15.172 D, d-GO/PVDF/ZnO 16.199 D), producing a dual dielectric benefit: (i) higher *ε*′ at low *f via* Maxwell–Wagner–Sillars interfacial polarization and microcapacitor-like charge storage, and (ii) higher σAC because σAC scales directly with *fε*′ tan *δ*, rationalizes the dielectric/impedance results by showing how GO chemistry and ZnO coupling progressively “activate” electronic states that are absent in pristine PVDF suggests that these materials possess enhanced electron transfer pathways, which are crucial for applications in electronic devices.^52^

### 3.6. Verification of the model studied

**Fig. 8:**
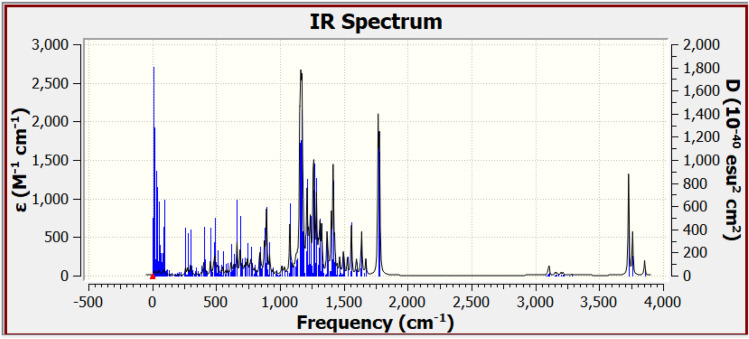
IR spectrum for d-GO/PVDF/ZnO which calculated at B3LYP/DGDZVP2.

As illustrated in Fig. 8, the FT-IR spectrum for the d-GO/PVDF/ZnO composite was calculated using the B3LYP/DGDZVP2 method. The absence of negative frequencies in the calculated spectrum suggests the stability of the optimized molecular structure, indicating the suitability of the model for the studied composite.^53–55^ This outcome affirms that the IR spectrum was derived from an optimized structure, underscoring the accuracy and reliability of the B3LYP/DGDZVP2 method. Further validation was performed through a comparison between the calculated IR spectrum and the experimental FT-IR data, as shown in Table 2. The results demonstrate a strong correlation between the calculated and experimental frequencies, confirming the robustness of the model studied. Specifically, the calculated IR frequencies closely match those obtained from FT-IR, supporting the accuracy of the computational approach.

**Table 2:** Comparison between FT-IR and calculated IR for d-GO/PVDF/ZnO which calculated at B3LYP/DGDZVP2

FTIR	Calculated	Assignment
484	488	CF_2_ bending
611	628	CF_2_ bending and skeletal bending modes
870	893	CH_2_ rocking
1673	1672	C=C sp^2^ hybridization of GO

### 3.7. Dielectric properties-frequency dependence

**Fig. 9:**
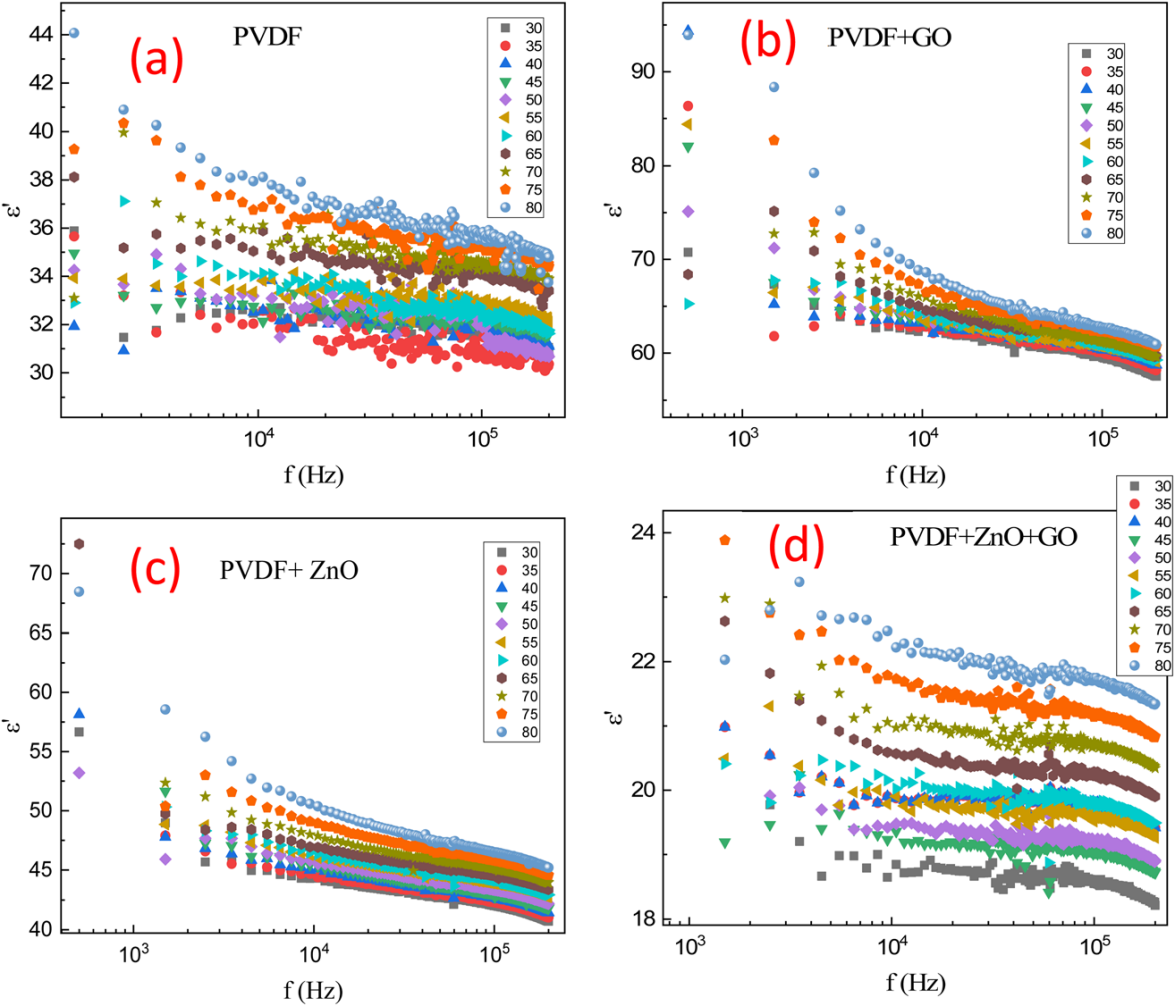
Dielectric constant (*ε*′) as a function of frequency (*f*) for (a) PVDF polymer, (b) PVDF/GO, (c) PVDF/ZnO, and (d) PVDF/ZnO/GO membranes at different temperature (30–80 °C).

Fig. 9 depicts the dielectric constant (*ε*′) as a function of frequency (*f*) for (a) PVDF polymer, (b) PVDF polymer doped with reduced graphene oxide (GO), (c) PVDF polymer doped with ZnO, and (d) PVDF polymer doped with ZnO/GO at various temperatures (30 °C to 80 °C).

The experimental results data in Fig. 9(a) presents the PVDF polymer. At lower frequencies, the dielectric constant is relatively high across all temperatures because all polarization mechanisms (electronic, ionic, dipolar, and interfacial) can effectively align with the applied electric field. As the temperature increases, the dielectric constant tends to be higher in the low-frequency region (*f* < 1000 Hz). While at higher temperatures (80 °C), the dielectric constant is greater than that at lower temperatures (30 °C), as elevated temperatures enhance the mobility of polymer chains and dipoles, allowing for better alignment and polarization. However, as the frequency increases (around 10^4^ Hz) the dielectric constant begins to decrease because of the slower polarization mechanisms (dipolar and interfacial), start lagging behind the rapidly changing electric field. Although further increases in temperature continue to produce higher dielectric constants compared to lower temperatures, the difference becomes less pronounced in the low-frequency region. At the highest frequencies (*f* > 10 kHz), the dielectric constant is lower for all temperatures, since only the fastest polarization mechanisms (electronic and ionic) can flow the high-frequency (electric field changes).

In the low-temperature range (30–50 °C), the dielectric constant remains relatively stable, with a slight increase observed due to improved dipolar alignment at lower frequencies (*f* < 10 kHz). As frequency increases, the dielectric constant decreases but remains slightly higher than at lower temperatures. At higher temperatures (50 °C < *T* < 80 °C), the dielectric constant shows a more pronounced increase at low frequencies (*f* < 10 kHz). While in the mid-frequency range (10 kHz < *f* < 100 kHz), the decrease is more significant, and at high frequencies (*f* > 100 kHz) the values begin to converge. This behavior indicates enhanced molecular mobility and polarization at elevated temperatures, reflecting maximum dipolar alignment. As the frequency further increases, the dielectric constant decreases sharply due to thermal agitation disrupting polarization, with the high-frequency values approaching those at lower temperatures as only electronic and ionic mechanisms contribute. These experimental observations suggest that PVDF is well-suited for applications such as sensors or capacitors operating at low frequencies and high temperatures, where a higher dielectric constant can enhance sensitivity and energy storage capacity. Conversely, the lower dielectric constant and its stability at high frequencies are beneficial for consistent performance in high-speed circuits and components.

Fig. 9(b) depicts the results of PVDF polymer doped with reduced graphene oxide (GO). The GO doped PVDF shows a generally higher dielectric constant compared to undoped PVDF due to the metallic behavior of the GO, which in turn makes nanoelectrods inside the samples and increased its total capacitance. This indicates that GO doping enhances the dielectric properties, likely due to increased interfacial polarization and improved dipole alignment. The decrease in dielectric constant with frequency is less steep in GO doped PVDF compared to undoped PVDF, suggesting that GO helps maintain higher polarization capabilities at higher frequencies. The temperature influence on the dielectric constant is more pronounced in GO doped PVDF at low frequencies. However, at high frequencies, the convergence of dielectric constants across temperatures indicates that GO does not significantly alter the high-frequency polarization mechanisms. The higher dielectric constant at low frequencies makes GO doped PVDF suitable for capacitors with higher energy storage capacity, efficiency, advanced medical sensors and implantable devices, piezoelectric sensors, actuators, which rely on the material's ability to polarize and depolarize efficiently, as well as the flexible electronic components and effective EMI shielding materials, protecting electronic devices from electromagnetic interference (providing stable performance across a range of frequencies and temperatures).

At frequencies (*f* < 10^3^ Hz), the dielectric constant is relatively high for all temperatures. This behavior is due to the contribution of all polarization mechanisms. For the higher temperatures (80 °C), the *ε*′ is higher compared to lower temperatures (30 °C). This indicates enhanced dipole mobility and interfacial polarization at raised temperatures and improved dipolar orientation with temperature. But with increasing frequency, the dielectric constant starts to decrease. The dipoles cannot follow the faster alternating electric field as efficiently. Rising temperatures (70–80 °C) show higher dielectric constants (65) than lower temperatures (55), though the difference reduces. At high frequencies (*f* > 100 kHz), the dielectric constant is lower across all temperatures. This is because only the electronic and ionic polarization can keep up with the high-frequency (electric field) changes. In addition, at different temperatures, the dielectric constant values converge more closely at higher frequencies, reflecting reduced temperature influence on the polarization mechanisms active at these frequencies. At higher temperature (80 °C), the dielectric constant is significantly higher at low frequencies, reflecting maximum dipolar alignment. As frequency increases, the dielectric constant decreases sharply, indicating more pronounced thermal agitation effects. At high frequencies, the dielectric constant values converge closely with those at lower temperatures.

Fig. 9(c) depicts the results of PVDF polymer doped with ZnO. The dielectric constant is relatively high across all temperatures, approximately the values of *ε*′ ranging from 55 to 70 because of all polarization mechanisms (electronic, ionic, dipolar, interfacial) can align with the electric field. The sample of ZnO doping exhibited higher dielectric constant compared to undoped PVDF, indicating enhanced dielectric properties due to increased polarization capabilities. Whiles, it is lower than that of GO doped PVDF, suggesting that GO has a more significant impact on enhancing the dielectric properties of PVDF. At higher temperatures (80 °C), the sample shows a higher *ε*′ compared to lower temperatures (30 °C). This suggests enhanced dipole mobility and interfacial polarization at elevated temperatures. with rising frequency, the dielectric constant decreases. The dipoles cannot flow the applied alternating electric field. Moreover, the higher temperatures show higher dielectric constants than lower temperatures, but the difference begins to diminish.

Fig. 9(d) shows the results of PVDF polymer doped with ZnO and reduced graphene oxide (GO). The dielectric constant decreases with increasing frequency, which is a common behavior in dielectric materials due to the inability of dipoles to follow the rapidly changing electric field at higher frequencies. At lower frequencies, the dielectric constant is higher, indicating greater polarization. Additionally, the experimental data points reveal that the dielectric constant generally decreases with increasing temperature, suggesting thermal agitation reduces the polarization efficiency within the material. The spread of data points for each temperature reflects this temperature-dependent behavior, with higher temperatures leading to a more pronounced decrease in dielectric constant at a given frequency. This trend signifies the interplay between thermal effects and frequency response in the dielectric properties of the doped PVDF polymer.

**Fig. 10:**
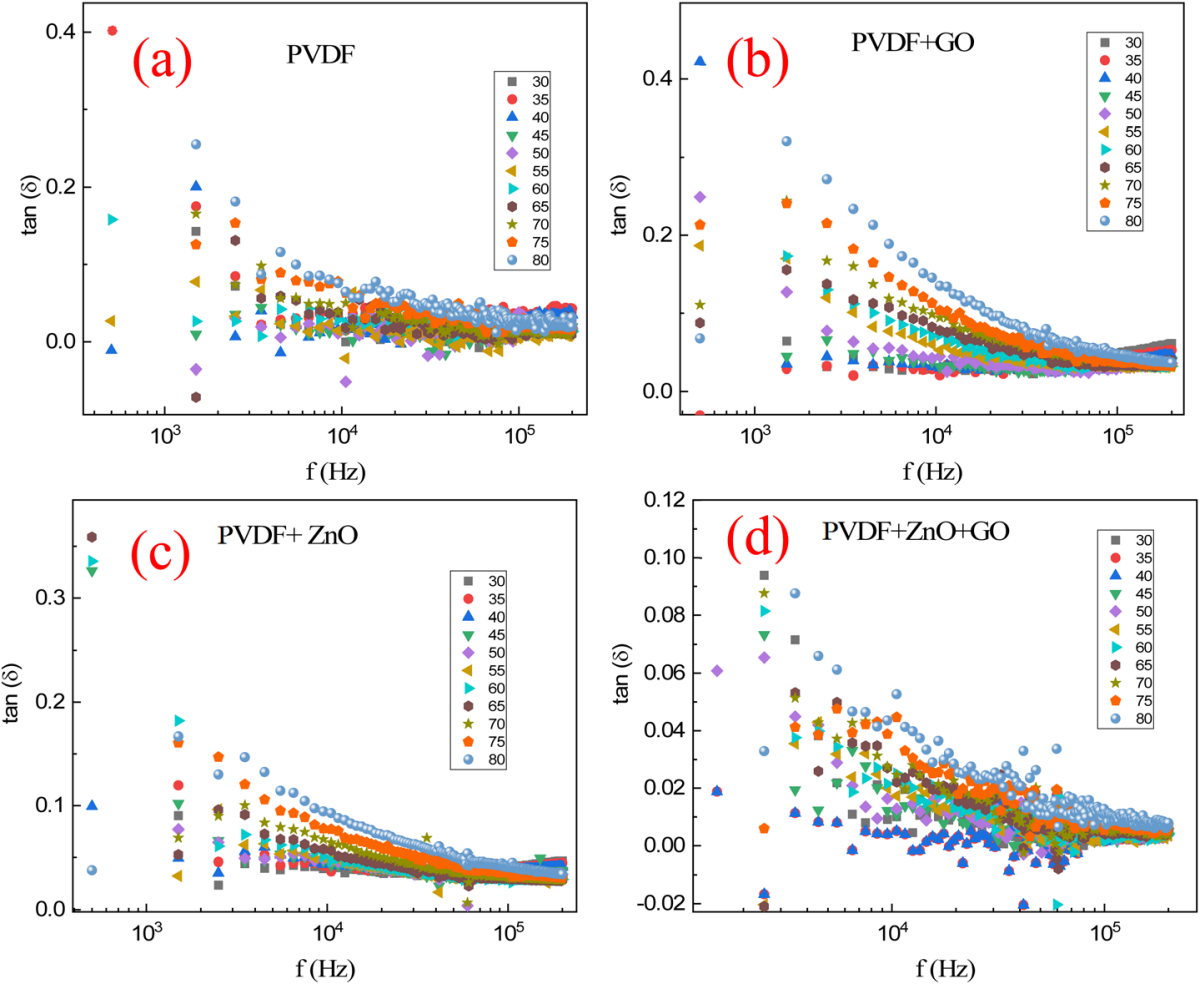
Dielectric tangent loss (tan *δ*) *versus* frequency for (a) PVDF polymer, (b) PVDF/GO, (c) PVDF/ZnO, and (d) PVDF/ZnO/GO membranes at different temperature (30–80 °C).

Fig. 10(a–d) shows the dielectric tangent loss (tan *δ*) *versus* frequency profiles for PVDF, GO-doped PVDF, ZnO-doped PVDF, and ZnO/GO co-doped PVDF polymers. The experimental results of the tangent loss (tan *δ*) reveals important information about the energy dissipation characteristics of these materials. The measurements were conducted over a temperature range from 30 °C to 80 °C, offering valuable insights into how doping with ZnO and GO affects the dielectric properties and energy loss mechanisms of the PVDF matrix under varying thermal conditions. For all samples, tan (*δ*) generally decreases with increasing frequency, indicating reduced energy dissipation at higher frequencies as dipole relaxation and charge carrier movement become less response for the rapidly changing electric field. The co-doped PVDF samples exhibits lower dielectric losses compared to its doped with the single filler, reflecting less internal friction and energy loss. The ZnO doping increases the dielectric loss due to the enhanced polarization and charge carrier activity introduced by the ZnO particles. Similarly, GO doping also increases dielectric loss, attributed to the conductive pathways and higher charge carrier mobility provided by the GO. The combination of ZnO and GO in the PVDF matrix results in the lowest dielectric losses, likely due to the synergistic effects of both dopants, which enhance polarization and charge carrier dynamics. Additionally, higher temperatures generally lead to increased dielectric losses for all samples at lower frequencies, due to intensified molecular motion and greater charge carrier mobility. However, this temperature effect diminishes at higher frequencies where the tan *δ* values converge, indicating a reduced impact of thermal agitation. This analysis highlights the complex interplay between doping, frequency, and temperature in determining the dielectric properties of PVDF-based composites.

**Fig. 11:**
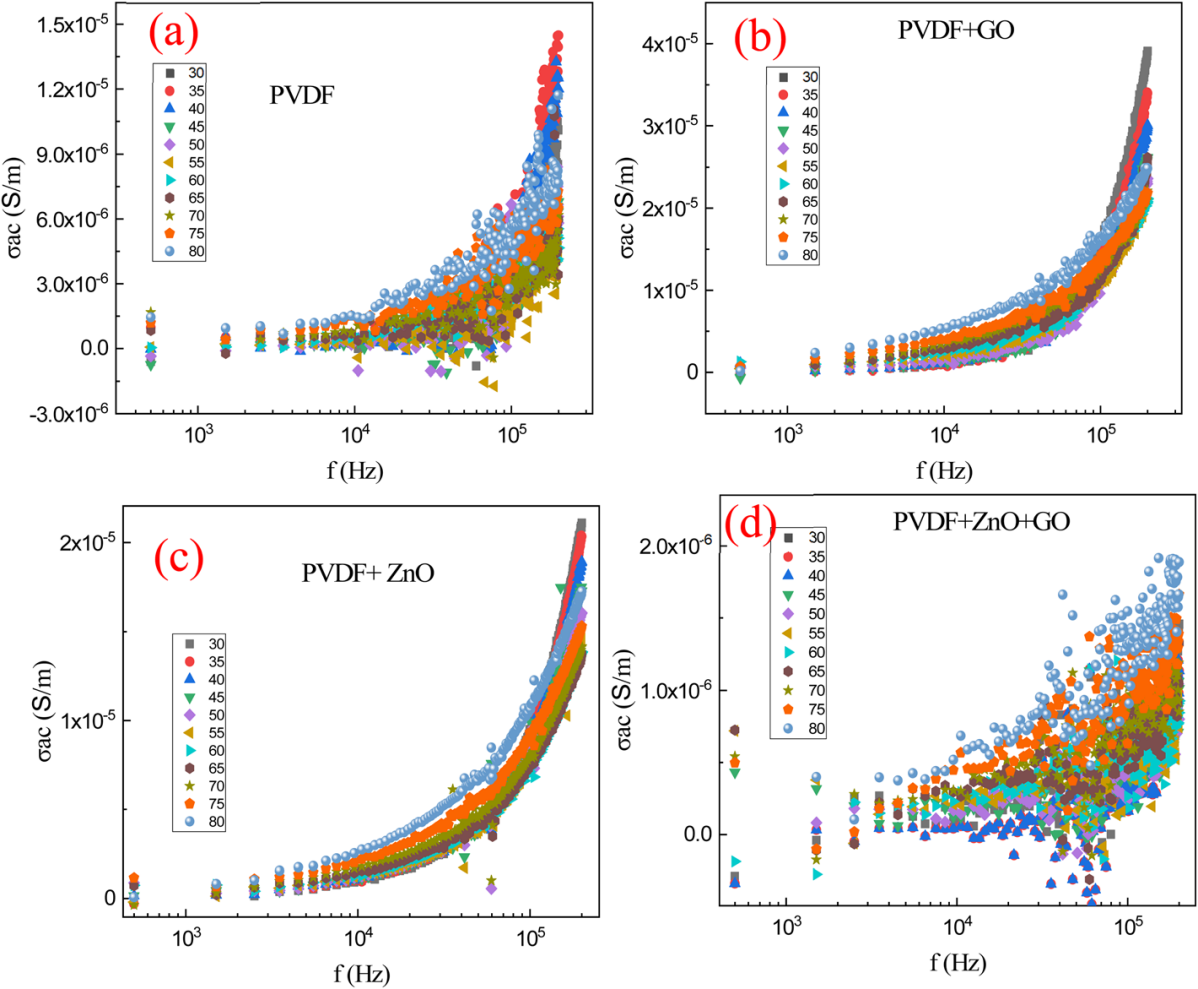
AC conductivity (*σ*_ac_) *versus* frequency relationship for (a) PVDF polymer, (b) PVDF/GO, (c) PVDF/ZnO, (d) PVDF/ZnO/GO membranes at different temperature (30–80 °C).

Fig. 11 depicts the AC conductivity (*σ*_ac_) *versus* frequency relationship for PVDF polymer, GO-doped PVDF polymer, ZnO-doped PVDF polymer, and ZnO/GO co-doped PVDF polymer. Whole the temperature ranges from 30 °C to 80 °C reveals crucial information about the charge transport mechanisms in these materials. Generally, AC conductivity increases with frequency for all samples, indicating a typical behavior where higher frequencies facilitate easier charge carrier movement. Pure PVDF shows the lowest conductivity due to its intrinsic insulating nature. But GO doping leads to a more significant increase in AC conductivity compared to ZnO doping alone, attributed to GO's excellent electrical conductivity and the formation of conductive networks within the polymer. While ZnO doping enhances the AC conductivity as ZnO particles introduce additional charge carriers and facilitate their movement through the polymer matrix. The co-doping of ZnO and GO in PVDF results in the highest AC conductivity, likely due to the combined effects of ZnO's charge carrier generation and GO's conductive pathways, significantly boosting the overall charge transport. Additionally, increasing the temperature generally enhances the AC conductivity for all samples, especially at lower frequencies, due to the increased thermal energy that promotes charge carrier mobility. However, at higher frequencies, the temperature effect on AC conductivity becomes less pronounced, indicating that frequency has a more dominant influence on charge transport dynamics. This behavior underscores the synergistic impact of frequency and temperature on the electrical properties of PVDF-based composites, particularly when modified with conductive and semi conductive dopants like ZnO and GO.

**Fig. 12:**
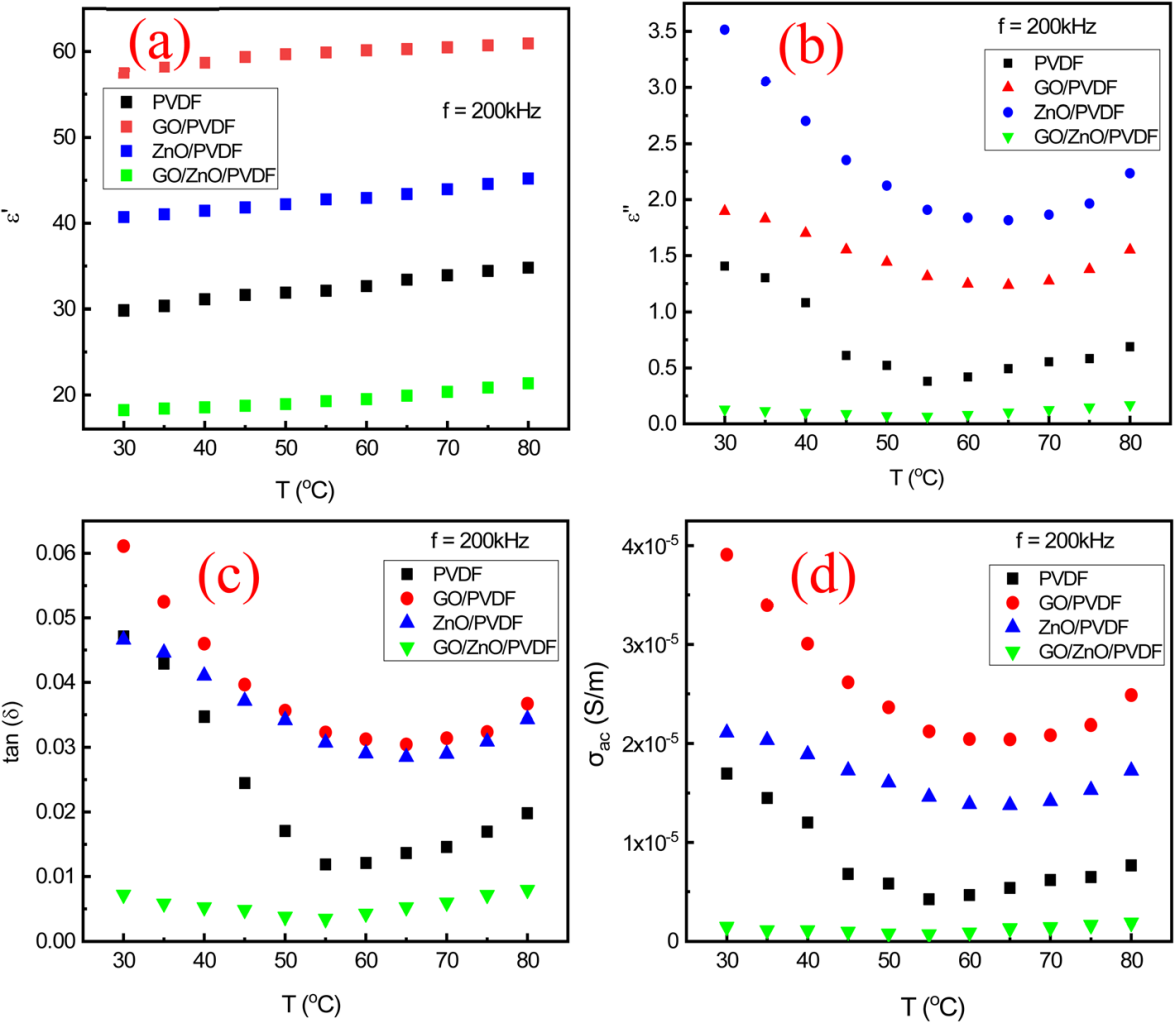
Relationship between dielectric loss and temperature for (a) PVDF polymer, (b) GO/PVDF, (c) ZnO/PVDF, and (d) ZnO/GO/PVDF membranes at a constant frequency (200 kHz).

Fig. 12(a) presented the relationship between the dielectric constant (*ε*′) and temperature for PVDF polymer, GO-doped PVDF polymer, ZnO-doped PVDF polymer, and ZnO/GO co-doped PVDF polymer at a constant frequency (200 kHz). These results reveals important insights into how temperature affects the polarization mechanisms in these materials. Generally, the dielectric constant tends to decrease with increasing temperature for all samples. In pure PVDF, this decrease is due to the reduced alignment of dipoles as thermal agitation increases, leading to lower polarization. In GO-doped PVDF, the dielectric constant is also elevated due to the high polarizability of GO and its conductive nature, which enhances interfacial polarization. Yet, as temperature rises, this enhancement diminishes due to increased thermal agitation. When ZnO is doped into PVDF, the dielectric constant is higher compared to pure PVDF, attributed to the added polarization from the ZnO particles. However, with increasing temperature, the dielectric constant still decreases as the thermal energy disrupts the alignment of the dipoles and the charge carriers contributed by ZnO. The ZnO/GO co-doped PVDF exhibits the highest dielectric constant among the samples, reflecting the combined effects of ZnO and GO in boosting polarization. Despite this, the dielectric constant of the co-doped sample also decreases with temperature, highlighting the overarching influence of thermal agitation in reducing dipole alignment and polarization. This behavior underscores the complex interplay between material composition, temperature, and dielectric properties, demonstrating that while dopants like ZnO and GO can enhance dielectric performance, elevated temperatures universally reduce the dielectric constant across all materials.

Fig. 12(b) depicted the relationship between dielectric loss and temperature for PVDF-based polymer composites of pure PVDF, GO-doped PVDF, ZnO-doped PVDF, and ZnO/GO-doped PVDF at a constant frequency (200 kHz). Dielectric loss, which measures the energy lost as heat due to dielectric polarization and resistive effects, generally increases with temperature due to enhanced molecular mobility and increased ionic conduction. For pure PVDF, dielectric loss tends to rise with temperature as thermal activation facilitates dipolar motion. The ZnO doping typically lowers dielectric loss at higher temperatures by reducing the polymer's resistive component, owing to ZnO's ability to stabilize the polymer matrix and improve charge carrier transport. On the other hand, GO doping can increase dielectric loss due to additional conductive pathways and increased interfacial polarization. When both ZnO and GO are combined in PVDF, the overall dielectric loss at elevated temperatures reflects a complex interplay between reduced resistive losses and enhanced polarization effects, making this composite particularly interesting for applications requiring optimized energy dissipation. Fig. 12(c) shows the relationship between dielectric tangent loss and temperature for PVDF-based polymers-namely, pure PVDF, GO-doped PVDF, ZnO-doped PVDF, and ZnO/GO-doped PVDF at a constant frequency (200 kHz) provides a clear picture of energy dissipation behaviors across different composites. Dielectric tangent loss, defined as the ratio of dielectric loss to the dielectric constant, quantifies the inefficiency of energy storage in a material. For pure PVDF, tangent loss typically increases with temperature due to enhanced dipolar relaxation and increased ionic conduction. In contrast, GO-doped PVDF often shows increased tangent loss at elevated temperatures due to additional conductive paths and increased interfacial polarization effects.

In ZnO-doped PVDF, tangent loss may decrease at higher temperatures because ZnO enhances charge transport and stabilizes the polymer matrix, reducing resistive losses. The ZnO/GO-doped PVDF composite generally exhibits complex behavior; the interplay between ZnO's stabilizing effects and GO's conductive properties results in a nuanced temperature-dependent tangent loss profile. This combined effect highlights the material's potential for applications requiring optimized dielectric performance and energy dissipation at varying temperatures.

Fig. 12(d) shows the relationship between AC conductivity (*σ*_ac_) and temperature for PVDF polymer, GO-doped PVDF polymer, ZnO-doped PVDF polymer, and ZnO/GO co-doped PVDF polymer at a constant frequency of 200 kHz reveals how temperature influences charge transport in these materials. As the temperature increases from 30 °C to 80 °C, the AC conductivity of all samples generally rises, reflecting enhanced thermal activation of charge carriers. For pure PVDF, the increase in conductivity is relatively modest due to its insulating nature and limited charge carrier mobility. ZnO doping significantly boosts the AC conductivity compared to pure PVDF, as ZnO particles introduce additional charge carriers and facilitate their movement. GO-doped PVDF shows an even more pronounced increase in conductivity with temperature, attributed to GO's excellent electrical conductivity and its ability to create conductive networks that improve charge transport. The combination of ZnO and GO in the PVDF matrix results in the highest AC conductivity, demonstrating a synergistic effect where ZnO provides more charge carriers and GO ensures efficient pathways for their movement. This combined effect is particularly evident at higher temperatures, where the thermal energy further enhances the mobility of charge carriers, leading to a steep rise in conductivity. The results shows that the analysis indicates that doping PVDF with ZnO and GO significantly improves its AC conductivity, especially at elevated temperatures, due to enhanced charge carrier generation and improved transport pathways.

Finally, in this study, the primary focus is a mechanistic investigation of how recycled carbon derived ZnO (Rc-ZnO) affects interfacial polarization, dielectric permittivity, and dielectric loss behavior in PVDF-based composite membranes. While the use of waste derived precursors is an important contextual element, the central contribution lies in elucidating the structure property relationships governing dielectric enhancement in these composites. Here, upcycling is defined as the transformation of waste-derived carbon precursors into functional nanostructured fillers with enhanced dielectric functionality. The dielectric constant and loss tangent of the PVDF/Rc-ZnO composites are compared with the PVDF-based dielectric systems, demonstrating competitive dielectric enhancement at relatively low filler loadings while maintaining controlled dielectric loss. These material characteristics specifically the combination of enhanced permittivity and acceptable loss support the relevance of the present composites for flexible dielectric layers in capacitive and energy-storage-related applications, thereby providing a clear link between the mechanistic findings and potential device relevance.

## 4 Conclusion

This study establishes a circular route to fabricate electroactive PVDF hybrid membranes by upcycling spent Zn–C batteries into carbon-doped recycled ZnO (Rc-ZnO) microspheres and integrating Rc-ZnO and/or graphene oxide (GO) into solution-cast PVDF. XRD/FT-IR confirm a filler-driven α → β phase enrichment *via* suppression of α-phase signatures and strengthening of β-sensitive bands indicating improved all-trans chain ordering that supports electroactivity. Electrically, dielectric measurements show frequency-dependent dispersion with enhanced interfacial polarization: GO and Rc-ZnO elevate permittivity compared with neat PVDF (*ε*′ reported ∼55–70 for ZnO-filled films), and ZnO/GO combination yields the highest AC conductivity with reduced loss relative to single-filler cases, reflecting synergistic carrier generation (ZnO) and conductive pathways (GO). DFT supports this mechanism by revealing a strong reduction in the electronic gap upon GO/ZnO interfacial coupling (PVDF: 9.251 eV *vs.* GO/PVDF/ZnO: 1.595 eV; d-GO/PVDF/ZnO: 0.833 eV) and a high dipole moment, consistent with activated polarization and facilitated charge transport. Future work should (i) standardize and report the β-phase fraction using an established FT-IR quantification protocol, and (ii) directly correlate composition with performance by mapping device-level and piezoelectric efficiency metrics (*e.g.*, *d*_33_, output voltage/current, and energy density) as a function of the Rc-ZnO/GO doping ratio.

### Ethical approval

This study does not involve human participants or animal subjects. Therefore, ethical approval was not required.

## Author contributions

I. Gomaa conceptualization, write and review the first draft and prepared the samples. F. Gamal, H. Kalil and H. Elhaes characterized the samples. A. I. Ali, D. Choi, and G. H. Ramzy measured and analyzed the dielectric properties. M. A. Ibrahim and H. Elhaes doing the DFT and DOS. I. Gomaa, M. A. Ibrahim, A. I. Ali, D. Choi write and review the manuscript.

## Conflicts of interest

We declare that there is no conflict of interest.

## Data Availability

All data are available upon reasonable request.

## References

[cit1] Ahbab N., Naz S., Xu T. B., Zhang S. (2025). A Comprehensive Review of Piezoelectric PVDF Polymer Fabrications and Characteristics. Micromachines.

[cit2] Saxena P., Shukla P. (2021). A Comprehensive Review on Fundamental Properties and Applications of Poly(Vinylidene Fluoride) (PVDF). Adv. Compos. Hybrid Mater..

[cit3] Turki O., Slimani A., Boufi S., Seveyrat L., Perrin V., Ben Hassen R., Khemakhem H. (2025). Sol-Gel Hydrothermal Synthesis of Lead-Free BT Nanoparticles for Enhanced Dielectric Properties in PVDF Nanocomposites. Appl. Surf. Sci..

[cit4] Prasad S., Chouhan H., Mushahary B. C., Chakravarty R., Parida B. N., Parida R. K. (2025). Exploring Optical, Dielectric and Transport Properties in PVDF Films for Advanced Applications. Mater. Chem. Phys..

[cit5] Rajwar S., Singh R. K., Singh S. K., Roy S. K., Prasad K. (2025). Tuning the β-Phase in BNBT/PVDF Composite Films: Structural and Impedance Study. J. Polym. Res..

[cit6] Shen Y., Li Z., Liu Y., Wang J. T. (2025). Impact of Hot Rolling on the Structure and Electrical Properties of Solution Cast PVDF Films and Its Optimization in Sensors. Chem. Eng. J..

[cit7] Chailad W., Yuennan J., Tohluebaji N., Nuchnong P., Tran Y., Martwong E., Yang L., Sukhawipat N. (2025). Silane-Modified Waste Amethyst as a Functional Filler in PVDF-HFP Composites for Flexible Dielectric and Energy Applications. J. Mater. Sci..

[cit8] Hajji M., Akkari A., Garcia-Loureiro A., Kamoun N. T. (2025). Bi and Bi-Ni Codoped CuO-ZnO Mixed Oxides: Advanced Catalysts for Efficient Rifampicin Degradation: Influence of Catalyst Dosage, PH, and Scavengers. Chem. Africa.

[cit9] Sanjay S. S., Pandey A. C., Ankit P., Chattopadhyaya M. C. (2013). Fabrication of Surfactant Sensing Membrane with ZnO Nano-Composite. Proc. Natl. Acad. Sci. India Sect. A Phys. Sci..

[cit10] Yu L., Xie Y., Liu M., Liu L., Chen Z., Yu Y. (2024). Piezoelectric PMS Activation by ZnO Embedded Polytetrafluoroethylene Membrane for Efficient Degradation of Carbamazepine. Desalin. Water Treat..

[cit11] Farzana R., Rajarao R., Behera P. R., Hassan K., Sahajwalla V. (2018). Zinc Oxide Nanoparticles from Waste Zn-C Battery via Thermal Route: Characterization and Properties. Nanomater.

[cit12] Vuong T. T. T., Nguyen P. L., Nguyen N. T., Phung T. V. B., Le P. A. (2024). Zinc-Carbon Battery Recycling for Investigating Carbon Materials for Supercapacitor Applications. ACS Omega.

[cit13] Abbas Z., Jahankhan M., Shin W. S., Jung S. M. G. (2025). Eco-Friendly Recycling and Conversion Technology of End-of-Life Alkaline Batteries into Electron Transport Layers for Organic Photovoltaic Solar Cells. Sep. Purif. Technol..

[cit14] Pattaweepaiboon S., Hirunpinyopas W., Iamprasertkun P., Pimphor K., Roddecha S., Dirayanti D., Boonchun A., Sirisaksoontorn W. (2024). Upcycling Electrode Materials from Spent Single-Use Zinc-carbon/Alkaline Batteries into Rechargeable Lithium-Ion Battery Application. J. Energy Storage.

[cit15] Saim A. K. (2024). Ammoniacal Leaching for the Extraction of Valuable Metals from Secondary Resources: A Review. Miner. Process. Extr. Metall. Rev..

[cit16] Aalami Z., Hoseinzadeh M., Hosseini Manesh P., Aalami A. H., Es’haghi Z., Darroudi M., Sahebkar A., Hosseini H. A. (2024). Synthesis, Characterization, and Photocatalytic Activities of Green Sol-Gel ZnO Nanoparticles Using Abelmoschus Esculentus and Salvia Officinalis: A Comparative Study versus Co-Precipitation-Synthesized Nanoparticles. Heliyon.

[cit17] Islam M. F., islam S., Miah M. A. S., Huq A. K. O., Saha A. K., Mou Z. J., Mondol M. M. H., Bhuiyan M. N. I. (2024). Green Synthesis of Zinc Oxide Nano Particles Using Allium Cepa L. Waste Peel Extracts and Its Antioxidant and Antibacterial Activities. Heliyon.

[cit18] Velenturf A. P. M., Purnell P. (2021). Principles for a Sustainable Circular Economy. Sustain. Prod. Consum..

[cit19] Adaval A., Chinya I., Bhatt B. B., Kumar S., Gupta D., Samajdar I., Aslam M., Turney T. W., Simon G. P., Bhattacharyya A. R. (2022). Poly (Vinylidene Fluoride)/Graphene Oxide Nanocomposites for Piezoelectric Applications: Processing, Structure, Dielectric and Ferroelectric Properties. Nano-Struct. Nano-Objects.

[cit20] Omar A., Gomaa I., Mohamed O. A., Magdy H., Kalloub H. S., Hamza M. H., Mohamed T. M., Rabee M. M., Tareq N., Hesham H. (2023). *et al.*, Investigation of Morphological, Structural and Electronic Transformation of PVDF and ZnO/RGO/PVDF Hybrid Membranes. Opt. Quant. Electron..

[cit21] Singh H. H., Khare N. (2018). Flexible ZnO-PVDF/PTFE Based Piezo-Tribo Hybrid Nanogenerator. Nano Energy.

[cit22] Shee C., Banerjee S., Bairagi S., Baburaj A., Naveen K. S. K., Aliyana A. K., Mulvihill D. M., Alagirusamy R., Ali S. W. (2024). A Critical Review on Polyvinylidene Fluoride (PVDF)/Zinc Oxide (ZnO)-Based Piezoelectric and Triboelectric Nanogenerators. J. Phys. Energy.

[cit23] Gomaa I., Hosny N. M., Elhaes H., Ezzat H. A., Elmahgary M. G., Ibrahim M. A. (2024). Two-Dimensional MXene as a Promising Adsorbent for Trihalomethanes Removal: A Density-Functional Theory Study. Nanomaterials.

[cit24] Gomaa I., Emam M. H., Wassel A. R., Ashraf K., Hussan S., Kalil H., Bayachou M., Ibrahim M. A. (2023). Microspheres with 2D RGO/Alginate Matrix for Unusual Prolonged Release of Cefotaxime. Nanomater..

[cit25] Gomaa I., Hosny N. M., Ibrahim M. A. (2024). Self-Assembled Dendrites of Graphene Oxide Quantum Dots via Bottom-up Lyophilization Synthesis. J. Mol. Struct..

[cit26] Kumbar S. S., Jarali C., Talange D. B., Kumbar R. B. (2020). Synthesis, Comparison and Analysis of Graphene. Mater. Today Proc..

[cit27] Ferella F., De Michelis I., Beolchini F., Innocenzi V., Vegliò F. (2010). Extraction of Zinc and Manganese from Alkaline and Zinc-Carbon Spent Batteries by Citric-Sulphuric Acid Solution. Int. J. Chem. Eng..

[cit28] Cerrillo-Gonzalez M. del M., Paz-Garcia J. M., Muñoz-Espinosa M., Rodriguez-Maroto J. M., Villen-Guzman M. (2024). Extraction and Selective Precipitation of Metal Ions from LiCoO2 Cathodes Using Citric Acid. J. Power Sources.

[cit29] Punt T., Akdogan G., Bradshaw S., van Wyk P. (2021). Development of a Novel Solvent Extraction Process Using Citric Acid for Lithium-Ion Battery Recycling. Miner. Eng..

[cit30] Aleid G. , Gomaa I., EL-Moslamy S. H., Alshammari A., Al-Marshedy S., Alshammary F., Abdel-Hameed R. and Kamoun E. A., Revolutionizing Antimicrobial Treatment: Unprecedented Synergy of Zno Quantum Dots/Ag Nps/Nitazoxanide Triumphs Composite Nps Over Multidrug-Resistant Human Pathogens, 10.2139/SSRN.4869391 PMC11450118 39361062

[cit31] Salam O. A., Hamad H. A., Eltokhy M. A. R., Ali A. I., Son J. Y., Ramzy G. H. (2024). A Comparative Study of PMMA/PEG Polymer Nanocomposites Doped with Different Oxides Nanoparticles for Potential Optoelectronic Applications. Sci. Rep..

[cit32] Morsy M., Gomaa I., Mokhtar M. M., ElHaes H., Ibrahim M. (2023). Design and Implementation of Humidity Sensor Based on Carbon Nitride Modified with Graphene Quantum Dots. Sci. Rep..

[cit33] Yogamalar R., Srinivasan R., Vinu A., Ariga K., Chandra Bose A. (2009). X-ray peak broadening analysis in ZnO nanoparticles. Solid State Commun..

[cit34] Raoufi D. (2013). Synthesis and microstructural properties of ZnO nanoparticles prepared by precipitation method Volume 50, February 2013, Pages 932-937. Renew. Energy.

[cit35] Mohan A. C., Renjanadevi B. (2016). Preparation of Zinc Oxide Nanoparticles and its Characterization Using Scanning Electron Microscopy (SEM) and X-Ray Diffraction(XRD). Procedia Technol..

[cit36] Abdullah I. Y., Jumali M. H. H., Yahaya M., Shanshool H. M. (2015). Facile Formation of β Poly (Vinylidene Fluoride) Films Using the Short Time Annealing Process. Adv. Environ. Biol..

[cit37] Satapathy S., Pawar S., Gupta P. K., RVarma K. B. (2011). Effect of Annealing on Phase Transition in Poly(Vinylidene Fluoride) Films Prepared Using Polar Solvent. Bull. Mater. Sci..

[cit38] Dhand V., Hong S. K., Li L., Kim J. M., Kim S. H., Rhee K. Y., Lee H. W. (2019). Fabrication of Robust, Ultrathin and Light Weight, Hydrophilic, PVDF-CNT Membrane Composite for Salt Rejection. Composites, Part B.

[cit39] Gupta A. K., Bajpai R., Keller J. M. (2008). PVDF: PI Nano Composite Films: Mechanical, FT-IR, XRD, AFM and Hydraulic Study. J. Polym. Res..

[cit40] Kumar S., Supriya S., Kar M. (2018). Enhancement of Dielectric Constant in Polymer-Ceramic Nanocomposite for Flexible Electronics and Energy Storage Applications. Compos. Sci. Technol..

[cit41] Arshad A. N., Wahid M. H. M., Rusop M., Majid W. H. A., Subban R. H. Y., Rozana M. D. (2019). Dielectric and Structural Properties of Poly(Vinylidene Fluoride) (PVDF) and Poly(Vinylidene Fluoride-Trifluoroethylene) (PVDF-TrFE) Filled with Magnesium Oxide Nanofillers. J. Nanomater..

[cit42] Xia Q., Zhao X. J., Chen S. J., Ma W. Z., Zhang J., Wang X. L. (2010). Effect of Solution-Blended Poly(Styrene-Co-Acrylonitrile) Copolymer on Crystallization of Poly(Vinylidene Fluoride). Express Polym. Lett..

[cit43] Prasad G., Liang J. W., Zhao W., Yao Y., Tao T., Liang B., Lu S. G. (2021). Enhancement of Solvent Uptake in Porous PVDF Nanofibers Derived by a Water-Mediated Electrospinning Technique. J. Mater..

[cit44] Bodhi T. K. . , K., Kurup H. P., Verma A., Karumuthil S. C. (2025). Synergistic Effect of MgO/ZnO on β-Phase Crystallization in PVDF: A Comparative Study of Mode of Nanofiller Additions for Piezoelectric Applications. Macromol. Chem. Phys..

[cit45] Davis J. L., Barteau M. A. (1990). Spectroscopic Identification
of Alkoxide, Aldehyde, and Acyl Intermediates in Alcohol Decomposition on Pd(111). Surf. Sci..

[cit46] Li W., Zhu Y. M., Wang G., Jiang B. (2016). Characterization of Coalification Jumps during High Rank Coal Chemical Structure Evolution. Fuel.

[cit47] Alam M. K., Sahadat Hossain M., Akash M. H., Miad A. Al, Bashar M. S., Bahadur N. M., Ahmed S. (2025). Morphological Change of ZnO Using Hydrothermal Technique and Organic Modifiers. Nano-Struct. Nano-Objects.

[cit48] Gomaa I., Hosny N. M., Ibrahim M. A. (2024). Self-Assembled Dendrites of Graphene Oxide Quantum Dots via Bottom-up Lyophilization Synthesis. J. Mol. Struct..

[cit49] Hester J. F., Mayes A. M. (2002). Design and Performance of Foul-Resistant Poly(Vinylidene Fluoride) Membranes Prepared in a Single-Step by Surface Segregation. J. Membr. Sci..

[cit50] Bayoumy A. M., Gomaa I., Elhaes H., Abdel-Aal M. S., Ibrahim M. A., Sleim M., Ibrahim M. A., Abdel-Aal M. S., Ibrahim M. A. (2021). Application of Graphene/Nickel Oxide Composite as a Humidity Sensor. Egypt. J. Chem..

[cit51] Alhuthali A. M. S., Kalil H., Ibrahim M. A. (2024). Evaluating the Reactivity of Polyvinyl Alcohol/Graphene Nanocomposites. Opt. Mater..

[cit52] Duan C. G., Mei W. N., Yin W. G., Liu J., Hardy J. R., Bai M., Ducharme S. (2003). Theoretical Study on the Optical Properties of Polyvinylidene Fluoridecrystal. J. Phys. Condens. Matter.

[cit53] Abdel Halim S., Ibrahim M. A. (2022). Synthesis, Structure Investigation, DFT Analysis, Optical, and Photoelectrical Properties of 9-Bromo-3-Hydroxychromeno[4,3-b]Pyrazolo[4,3-e]Pyridin-5(1H)-One (BHCPP). Results Chem..

[cit54] El-Demerdash S. H., Halim S. A., El-Nahas A. M., El-Meligy A. B. (2023). A Density Functional Theory Study of the Molecular Structure, Reactivity, and Spectroscopic Properties of 2-(2-Mercaptophenyl)-1-Azaazulene Tautomers and Rotamers. Sci. Rep..

[cit55] El-Saady A. A., Roushdy N., Farag A. A. M., El-Nahass M. M., Abdel Basset D. M. (2023). Exploring the Molecular Spectroscopic and Electronic Characterization of Nanocrystalline Metal-Free Phthalocyanine: A DFT Investigation. Opt. Quant. Electron..

